# Transition zone prostate cancer is associated with better clinical outcomes than peripheral zone cancer

**DOI:** 10.1002/bco2.47

**Published:** 2020-10-06

**Authors:** Shun Sato, Takahiro Kimura, Hajime Onuma, Shin Egawa, Hiroyuki Takahashi

**Affiliations:** ^1^ Department of Pathology the Jikei University School of Medicine Tokyo Japan; ^2^ Department of Urology the Jikei University School of Medicine Tokyo Japan

**Keywords:** biochemical recurrence, clinical progression, ERG, prostate cancer, PTEN, transition zone

## Abstract

**Objectives:**

To determine the biological significance of zonal origins in prostate cancer.

**Patients and methods:**

Altogether, 270 consecutive radical prostatectomy cases from 2009 to 2012 were adopted. Cases were divided into those having transition zone (TZ) cancer or peripheral zone (PZ) cancer. Cases with indeterminate tumor location and central zone cancers were excluded from the analyses. Prognosis and clinicopathological features were compared between the two tumor locations. Biochemical recurrence (BCR) and clinical progression (CP) were adopted as prognostic outcome measures. Immunohistochemical features of the v‐ets avian erythroblastosis virus E26 oncogene homolog (ERG)/serine peptidase inhibitor, Kazal‐type 1 (SPINK1) status, and loss of phosphatase and tensin homolog (PTEN‐loss), as well as conventional preoperative and postoperative characteristics, were analyzed.

**Results:**

This cohort comprised 93 cases of TZ cancer and 160 cases of PZ cancer. TZ cancer cases showed significantly higher BCR and CP‐free survival rate than PZ cancer cases. Notably, no TZ cancer cases developed CP during the 7.8 years of median follow‐up time. Tumor location was an independent predictive factor for BCR in the multivariate analysis. Additionally, TZ cancer cases showed a significantly lower prevalence of ERG‐overexpression and PTEN‐loss than PZ cancer cases (3.2% vs 20.1% and 2.2% vs 18.2%, respectively).

**Conclusion:**

TZ cancer cases showed a better prognosis and different immunohistochemical features. Conservative treatment strategies could be considered for TZ cancer cases.

## 1 INTRODUCTION

The incidence of prostate cancer (PCa) has dramatically increased since the introduction of prostate‐specific antigen (PSA) screening in the late 1980s. Significant variations have been reported for the incidence of PCa between races and regions. For instance, Asian countries are known to have a low incidence, which is one‐tenth compared to the highest incidence in European countries,^1^ although Asian countries are known for their high mortality‐to‐incidence ratios associated with PCa.^2^ These differences are partly explained by the delay of the introduction of a large‐scale PSA screening program in most Asian countries. Moreover, differences in the anatomical location [eg, peripheral zone (PZ) vs transition zone (TZ)] and genetic alteration among races and regions might affect the difference in PCa incidence and/or mortality‐to‐incidence ratio.

McNeal proposed the anatomical classification of the prostate in the late 1960s, which has been widely used for clinicopathological studies of the prostate.^3^ He divided the prostate gland into three segments as follows: the TZ surrounding the proximal prostatic urethra, the PZ located on the posterolateral to apical prostate, and the central zone (CZ) located from the verumontanum to the posterobasal region along the ejaculatory ducts.^3^, ^4^ Several studies from Western countries have shown that the majority of PCa arose in the PZ, and cancer in the TZ was less frequent (4%‐20%).^5^, ^6^, ^7^, ^8^ However, our previous study showed that the prevalence of TZ cancer was as much as 42% in a Japanese cohort, and subsequent studies from eastern Asian countries also showed a higher prevalence of TZ cancers (30%‐46%) than those from Western countries.^9^, ^10^, ^11^, ^12^, ^13^

Although the genetic features of PCa remain unclear to date, the racial differences associated with the most frequent genetic alterations of the transmembrane protease, serine 2 (TMPRSS2)‐v‐ets avian erythroblastosis virus E26 oncogene homolog (ERG) gene fusion and phosphatase and tensin homolog (PTEN) inactivation have been reported. The prevalence of the TMPRSS2‐ERG gene fusion/ERG‐overexpression in PCa was approximately 50% in Western countries,^14^, ^15^, ^16^ although the prevalence is less at 10%‐20% in east‐Asian countries.^16^, ^17^ Recent studies reported an association between the TMPRSS2‐ERG gene fusion and another well‐known prognostic marker, serine peptidase inhibitor, Kazal‐type 1 (SPINK1). Because ERG and SPINK1 show exclusive expression, some studies divided PCa into three categories as follows: ERG‐/SPINK1‐(double negative), ERG‐/SPINK1+, and ERG+/SPINK1‐ by ERG/SPINK1 status.^18^, ^19^, ^20^ The SPINK1‐positive cancer cases were reportedly associated with poorer outcomes among ERG‐negative cancer cases. A few studies on the prevalence of PTEN inactivation have shown less prevalence in the African American and Asian populations than in the Caucasian population.^2^, ^16^

Recent advances in PCa diagnosis, such as multiparametric magnetic resonance imaging (MRI) and MRI‐ultrasound fusion targeted biopsy, have enabled clinicians to diagnose PCa location without surgery. Here, the knowledge of the biological features of PCa of different zonal origins is crucial for the daily practice of PCa management, especially for selecting treatment options such as focal therapies. Previous reports including our research have shown that clinicopathological differences exist between TZ and PZ cancers. In these studies, TZ cancer cases showed a lower grade and earlier stage than PZ cancer cases.^7^, ^9^, ^11^, ^21^ However, differences in the oncological outcomes and molecular characteristics between both cancers have not been well‐elucidated. Here, we conducted further examinations, including prognostic and immunohistochemical analyses, to show the detailed biological differences between both cancers.

## 2 PATIENTS AND METHODS

### 2.1 Patients

Altogether, 270 consecutive radical prostatectomy (RP) cases diagnosed by a single genitourinary pathologist (HT) from August 2009 to August 2012 were adopted. Cases with any preoperative treatment, such as endocrine therapy, radiation therapy, and chemotherapy, were excluded. Eighteen cases with indeterminate cancer location or CZ location were excluded from statistical analyses as mentioned below. Hence, 252 cases were ultimately analyzed.

### 2.2 Pathological examination

The RP specimens were routinely processed, and pathological diagnosis was made as previously reported.^9^ Cases were divided into those having TZ or PZ cancers based on their tumor location. The TZ and PZ were defined microscopically according to the McNeal's criteria.^3^, ^4^ The zonal origin of the tumor was determined using a tumor mapping picture written by microscopic examination. In the multifocal cases, the index tumor was utilized for analyses. The index tumor was defined as the largest tumor in the case, similar to the definition used in previous studies.^8^, ^11^, ^22^ In the current study, tumors extending over the TZ and PZ were treated as cancers of no dominant location (ND) and were excluded from statistical analyses. Thus, the definition of TZ/PZ cancer is rather different from that in our previous study.^10^ The CZ cancer cases were also excluded from the analyses.

In the RP specimens, intraductal carcinoma was examined by one genitourinary pathologist (SS) according to the McNeal's criteria.^23^

Immunohistochemical analyses for anti‐ERG rabbit monoclonal antibody (clone EPR 3,864, Abcam, Cambridge, UK), anti‐PTEN rabbit monoclonal antibody (clone D4.3, Cell Signalling Technology, Denvers, MA, US), and anti‐SPINK1 mouse monoclonal antibody (clone 4D4, Abnova, Taipei City, Taiwan) were performed using BenchMark XT automated stainer (Roche Diagnostics K.K., Tokyo, Japan). In the immunohistochemical analyses, the cut‐off value was set as 10%. Thus, cases were categorized as ERG/SPINK1 positive or PTEN‐loss if 10% or more of tumor cells stained for ERG/SPINK1 antibody or lost the staining for PTEN antibody.

### 2.3 Acquisition of clinical information

For each patient, clinical information such as age, serum PSA level, clinical T stage, and postoperative follow‐up data, were collected from medical records.

### 2.4 Outcome measures

We adopted biochemical recurrence (BCR) and clinical progression (CP) as the primary and secondary measures of treatment outcome, respectively. The definition of BCR was as previously described.^24^ The PSA cut‐off value for BCR was set as 0.2 ng/mL. If postoperative treatment (eg, endocrine and radiation therapy) was initiated in the absence of an increase in the serum PSA level to ≥0.2 ng/mL, the day of BCR was defined as the day of initial treatment. CP was defined as metastasis detected by radiological examination including bone scintigraphy. In our institute, treatment after biochemical recurrence is uniformly provided to the patients as follows; salvage radiation therapy with 65 Gy or more with/without androgen deprivation therapy on BCR after RP; androgen deprivation therapy on the second BCR after salvage radiation therapy.

### 2.5 Statistical analyses

In the first analysis, we compared the clinicopathological features and prognoses between TZ and PZ cancers. The Mann‐Whitney U‐test was performed for comparing continuous (numerical) variables, and the Fisher's exact test was used for categorical variables. BCR‐free and CP‐free survival rates were calculated by the Kaplan–Meier method, and the inter‐group difference was compared by the log‐rank test. Additionally, multivariate Cox regression analysis for BCR was performed to examine the prognostic value of tumor location. Different multivariate models were assigned for preoperative and postoperative features (Tables 3 and 4, respectively). The preoperative features were age, PSA, clinical T stage (cT), Grade Group (GG) in the biopsy specimen, and percentage of the positive core. The postoperative features were tumor volume, GG in RP specimen, pathological T stage (pT), surgical margin status, lymph node metastasis, ERG/SPINK1 status, and PTEN‐loss. Tumor location (TZ vs PZ) was included in both models.

Thereafter, we examined the clinicopathological significance of ERG/SPINK1 expression status in each tumor location. The TZ and PZ cancers were subdivided into three categories by ERG/SPINK1 status as ERG‐/SPINK1‐, ERG‐/SPINK1+, and ERG+/SPINK1‐, as previously reported.^20^ Clinicopathological and prognostic (BCR) differences were compared between the groups. In the TZ group, ERG+/SPINK1‐ cases were excluded from statistical analyses because of extremely low prevalence (see Results). Additionally, the Kruskal‐Wallis test was used to examine the inter‐group difference of continuous variables among the three groups.

In all the analyses, statistical significance was defined as a *P*‐value of <.05. The EZR software package based on R (R Foundation for Statistical Computing, Vienna, Austria) was used for the analyses.^25^

### 2.6 Research ethics

This study received prior approval from the Ethics Committee of the affiliated institution (institutional ID number: 25‐312). Informed consent was not required due to the retrospective study design.

## 3 RESULTS

### 3.1 Clinicopathological patient backgrounds

This cohort comprised 93 cases (34.4%) of TZ cancer and 159 cases (58.9%) of PZ cancer. The clinicopathological patient backgrounds are shown in Tables 1 and 2. Regarding preoperative features, significant differences were shown in cT, GG in the biopsy specimen, and percentage of the positive core. TZ cancer cases included more cTlc cancers and showed lower GG and percentage of positive core than PZ cancer cases. Accordingly, TZ cancer cases showed lower National Comprehensive Cancer Network (NCCN) risk group. A significant difference was not observed for age and PSA in both groups. In addition, we compared the relationship between the history of preoperative MRI examination and tumor location. Altogether, 97 cases (35.9%) underwent preoperative MRI examination, of which 36 cases (37.1%) had TZ cancer. Moreover, 173 cases (64.1%) did not undergo an MRI examination before surgery, of which 57 cases (32.9%) had TZ cancer. The prevalence of TZ cancer was not significantly different between patients with and without preoperative MRI examination (*P* = .587).

**TABLE 1:** Preoperative characteristics

Zonal location	Whole cohort	TZ cancer	PZ cancer	ND and CZ cancer	*P*‐value*
Case number (%)	270 (100)	93 (34.4)	159 (58.9)	18 (6.7)	N/A
Age	65 (61‐69)	64 (60‐69)	66 (61‐70)	66 (60‐68.6)	.255
PSA (ng/mL)	8.15 (5.80‐11.79)	7.66 (5.70‐11.57)	8.26 (5.95‐11.31)	14.09 (6.19‐24.17)	.502
<10 (%)	172 (63.7)	65 (69.9)	100 (62.9)	7 (38.9)	.271
10‐20 (%)	72 (26.7)	19 (20.4)	47 (29.6)	6 (33.3)	
>20 (%)	26 (9.6)	9 (9.7)	12 (7.5)	5 (27.8)	
Clinical T stage					
T1c (%)	108 (40.0)	43 (46.2)	59 (37.1)	6 (33.3)	.0265^a^
T2a (%)	82 (30.4)	29 (31.2)	47 (29.6)	6 (33.3)	
T2b (%)	40 (14.8)	10 (10.8)	28 (17.6)	2 (11.1)	
T2c (%)	18 (6.7)	9 (9.7)	8 (5.0)	1 (5.6)	
T3a (%)	17 (6.3)	2 (2.2)	14 (8.8)	1 (5.6)	
T3b (%)	5 (1.9)	0 (0)	3 (1.9)	2 (11.1)	
Grade group in biopsy					
Grade Group 1 (%)	66 (24.4)	33 (35.5)	29 (18.2)	4 (22.2)	.0008
Grade Group 2 (%)	91 (33.7)	31 (33.3)	53 (33.3)	7 (38.9)	
Grade Group 3 (%)	41 (15.2)	17 (18.3)	24 (15.1)	0 (0)	
Grade Group 4 (%)	42 (15.6)	5 (5.4)	33 (20.8)	4 (22.2)	
Grade Group 5 (%)	30 (11.1)	7 (7.5)	20 (12.6)	3 (16.7)	
% Positive core	30 (16.7‐50)	16.7 (12.3‐34.1)	33.3 (25‐50)	51.9 (25.6‐76.7)	<.0001
History of negative biopsy (%)	30 (11.1)	17 (18.3)	10 (6.3)	3 (16.7)	.0052
NCCN risk classification					
Very low/low (%)	49 (18.2)	26 (28.0)	21 (13.2)	2 (11.1)	.0043
Intermediate (%)	125 (46.3)	46 (49.5)	72 (45.3)	7 (38.9)	
High (%)	78 (28.9)	18 (19.4)	55 (34.6)	5 (27.8)	
Very high (%)	18 (6.7)	3 (3.2)	11 (6.9)	4 (22.2)	

Note

Continuous variables are shown in median value and interquartile range.

^a^ Cases are categorized into T1c/2a, 2b/2c, and 3a/3b for statistical analysis.

* *P*‐values are calculated by comparison of TZ and PZ cancer.

**TABLE 2:** Pathological features in radical prostatectomy specimen

Zonal location	Whole cohort	TZ cancer	PZ cancer	ND and CZ cancer	*P*‐value*
Case number (%)	270 (100)	93 (34.4)	159 (58.9)	18 (6.7)	N/A
Prostate volume (cm^3^)	185.40 (143.86‐231.59)	172.95 (134.52‐240.21)	187.93 (147.69‐228.32)	181.16 (156.09‐257.92)	.3802
Tumor volume (cm^3^)	2.70 (0.99‐5.89)	2.56 (1.21‐5.02)	2.76 (0.81‐5.89)	7.17 (2.48‐14.97)	.593
Grade group					
Grade Group 1 (%)	39 (14.4)	22 (23.7)	14 (8.8)	3 (16.7)	.0011
Grade Group 2 (%)	126 (46.7)	49 (52.7)	71 (44.7)	6 (33.3)	
Grade Group 3 (%)	48 (17.8)	11 (11.8)	34 (21.4)	3 (16.7)	
Grade Group 4 (%)	10 (3.7)	2 (2.2)	6 (3.8)	2 (11.1)	
Grade Group 5 (%)	47 (17.4)	9 (9.7)	34 (21.4)	4 (22.2)	
Extraprostatic extension (EPE)					
EPE0 (%)	112 (41.5)	48 (51.6)	60 (37.7)	4 (22.2)	.004
EPE1 (%)	123 (45.6)	29 (31.2)	83 (52.2)	11 (61.1)	
EPEx (%)^a^	35 (13.0)	16 (17.2)	16 (10.1)	3 (16.7)	
Pathological T stage					
pT2 (%)	147 (54.4)	64 (68.8)	76 (47.8)	7 (38.9)	<.0001
pT3a (%)	83 (30.7)	29 (31.2)	48 (30.2)	6 (33.3)	
pT3b (%)	40 (14.8)	0 (0)	35 (22.0)	5 (27.8)	
Positive surgical margin (%)	96/270 (35.6)	29/93 (31.2)	54/159 (34)	5/18 (72.2)	.679
Intraductal carcinoma (%)	92/270 (34.1)	10/93 (10.8)	71/159 (44.7)	11/18 (61.1)	<.0001
Lymph node metastasis (pN)					
pN0 (%)	232 (85.9)	85 (91.4)	134 (84.3)	13 (72.2)	.186
pN1 (%)	21 (7.8)	3 (3.2)	14 (8.8)	4 (22.2)	
pNx (%)	17 (6.3)	8 (5.4)	11 (6.9)	1 (5.6)	
ERG overexpression (%)	40 (14.8)	3/93 (3.2)	32/159 (20.1)	5/18 (27.8)	.0001
PTEN‐loss (%)	35 (13.0)	2/93 (2.2)	29/159 (18.2)	4/18 (22.2)	<.0001
SPINK1 expression (%)	42 (15.6)	15/93 (16.1)	24/159 (15.1)	3/18 (16.7)	.858
Subgroup by the combination of ERG and SPINK1 expression					
ERG‐/SPINK1− (%)	188 (69.6)	75 (80.6)	103 (64.8)	10 (55.6)	.0003
ERG‐/SPINK+ (%)	42 (15.6)	15 (16.1)	24 (15.1)	3 (16.7)	
ERG+/SPINK− (%)	40 (14.8)	3 (3.2)	32 (20.1)	5 (27.8)	

Note

Continuous variables are shown in median value and interquartile range.

^a^ EPEx means indeterminate EPE status because of surgical incision into intraglandular cancer.

* *P*‐values are calculated by comparison of TZ and PZ cancer.

Regarding postoperative pathological findings, TZ cancer cases showed significantly lower GG and pT than PZ cancer cases. Lymph node metastasis was detected more frequently in PZ cancer cases (8.8%) than in TZ cancer cases (3.2%), although a significant difference was not shown (*P* = .186). Moreover, no significant difference was shown for tumour volume and positive surgical margin rate. The immunohistochemical profile in the entire cohort was as follows: ERG‐overexpression shown in 14.8%, PTEN‐loss in 13.0%, and SPINK1 expression in 15.6%. Compared to PZ cancer cases, TZ cancer cases showed an extremely lower prevalence of ERG‐overexpression (3.2% vs 20.1%) and PTEN‐loss (2.2% vs 18.2%) with a significant difference (*P* = .0001 and <.0001, respectively). A significant difference was not shown for SPINK1 expression (*P* = .858).

### 3.2 Tumor location and biochemical recurrence

**FIGURE 1:**
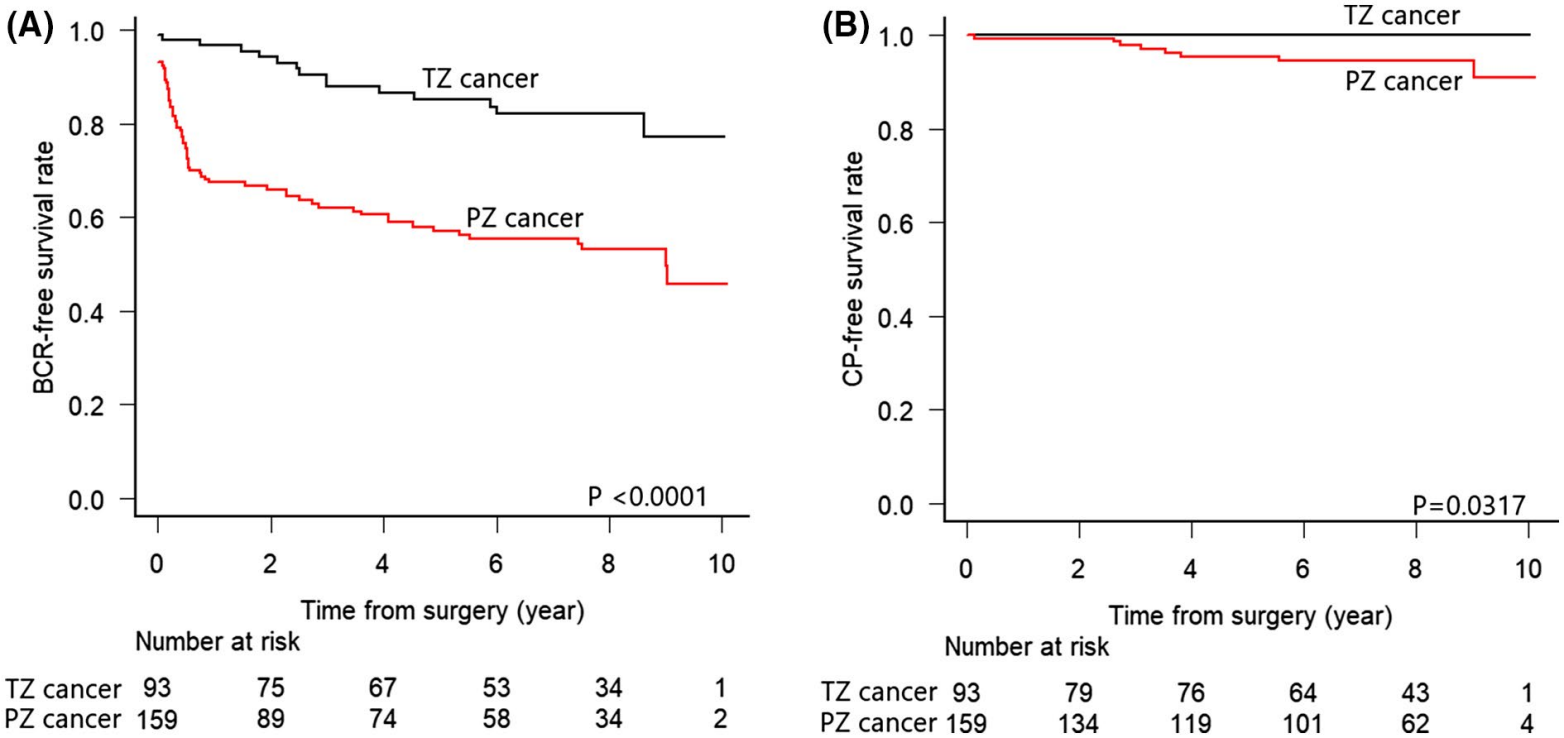
Kaplan–Meier curves for BCR (a) and CP (b)‐free survival rates stratified by tumor locations

The median follow‐up period was 93.8 (interquartile range: 59.3‐102) months in the entire cohort, 93.4 (60‐101.8) months in TZ cancer cases, and 93.6 (55.3‐101.9) months in PZ cancer cases without a significant difference (*P* = .854). TZ cancer cases showed a significantly higher BCR‐free survival rate than PZ cancer cases (*P* < .0001, Figure 1a). The results for univariate and multivariate Cox regression analyses are shown in Tables 3 and 4. In the multivariate model incorporating preoperative factors and tumor location, tumor location was an independent predictive factor of BCR (*P* = .0008), as well as PSA, cT, and GG in biopsy (Table 3). In the multivariate model incorporating postoperative pathological features and tumor location, tumor location was an independent predictive factor of BCR (*P* = .036), as well as GG in RP specimen, positive surgical margin, and lymph node metastasis (Table 4). PTEN‐loss was associated with an increased risk of BCR in the univariate analysis (*P* = .0021), although it was not an independent predictive factor in the multivariate analysis (*P* = .2585). The ERG/SPINK1 status was not predictive of BCR in both the univariate and multivariate analyses in the entire cohort.

**TABLE 3:** Cox regression analysis for BCR; comparison of preoperative factors and tumor location

	Univariate		Multivariate	
	HR (95% CI)	*P*‐value	HR (95% CI)	*P*‐value
Age	1.018 (0.986‐1.05)	.2773		
PSA	1.063 (1.045‐1.082)	<.0001	1.051 (1.023‐1.080)	.0004
cT (vs cT1c/2a)				
cT2b/2c	1.983 (1.239‐3.174)	.0043	1.690 (1.024‐2.789)	.0403
cT3	6.281 (3.649‐10.810)	<.0001	3.731 (1.953‐7.127)	<.0001
Grade group in biopsy (vs GG1/2)				
Grade group 3‐5	2.926 (1.939‐4.413	<.0001	2.441 (1.542‐3.866)	.0001
% Positive core	1.02 (1.012‐1.028)	<.0001	1.005 (0.995‐1.016)	.3254
PZ tumor location (vs TZCa)	3.471 (1.985‐6.068)	<.0001	2.834 (1.568‐5.121)	.0006

**TABLE 4:** Cox regression analysis for BCR; comparison of postoperative pathological features and tumor location

	Univariate		Multivariate	
	HR (95% CI)	*P*‐value	HR (95% CI)	*P*‐value
Tumor volume^a^	1.105 (1.075‐1.137)	<.0001	1.027 (0.983‐1.073)	.2323
Grade Group (vs GG1/2)				
Grade group 3‐5	4.708 (3.08‐7.196)	<.0001	3.593 (2.089‐6.181)	<.0001
pT (vs pT2) pT3	3.91 (2.527‐6.051)	<.0001	1.649 (0.924‐2.944)	.0908
Positive surgical margin	2.598 (1.741‐3.875)	<.0001	1.794 (1.113‐2.890)	.0164
Lymph node metastasis (vs pN0/x) pN1	9.395 (5.527‐15.97)	<.0001	3.780 (1.881‐7.598)	.0002
Intraductal carcinoma	4.87 (3.217‐7.372)	<.0001	1.288 (0.704‐2.357)	.4118
PTEN‐loss	2.208 (1.335‐3.655)	.0021	1.496 (0.807‐2.770)	.2006
Subgroup by the combination of ERG and SPINK1 overexpression (vs ERG‐/SPINK1‐)				
ERG‐/SPINK1+	1.074 (0.622‐1.855)	.7969		
ERG+/SPINK−	1.080 (0.616‐1.893)	.7882		
PZ tumor location (vs TZCa)	3.471 (1.985‐6.068)	<.0001	2.241 (1.214‐4.136)	.0099

^a^ HR shows risk by a tumor volume increase of 1 cm^3^.

### 3.3 Tumor location and clinical progression

TZ cancer cases showed a significantly higher CP‐free survival rate than PZ cancer cases (*P* = .0317, Figure 1b). Notably, no TZ cancer case developed CP during the median follow‐up period of 7.8 years, although eight cases of PZ cancer and 11 cases in the entire cohort developed CP.

### 3.4 Clinicopathological significance of ERG/SPINK1 status in transition zone cancers

**FIGURE 2:**
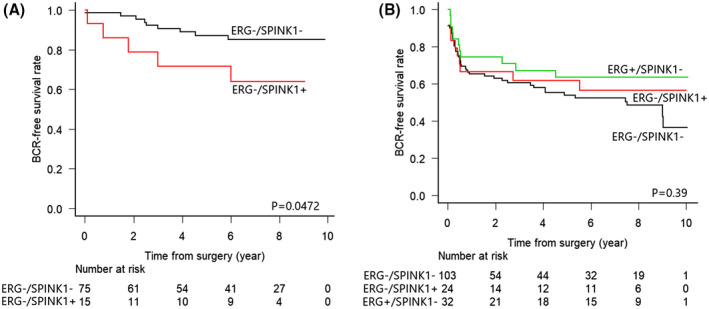
Kaplan–Meier curves for BCR in TZ (a) and PZ (b) cases stratified by ERG/SPINK1 status. In both figures, the black line shows ERG‐/SPINK1‐cases, the red line shows ERG‐/SPINK1 + cases, and the green line shows ERG+/SPINK1‐cases

In the TZ cancer group, statistical analyses were conducted only between ERG‐/SPINK1‐ and ERG‐/SPINK1 + subgroups because of the extremely lower prevalence of ERG‐overexpression in this group. The ERG‐/SPINK1 + cases showed a significantly lower BCR‐free survival rate (*P* = .0472, Figure 2a), although no clinicopathological parameters showed a significant difference between the groups (Table 5).

**TABLE 5:** Clinicopathological comparison by ERG/SPINK1 status in TZ/PZ cancer

	Transition zone cancer			Peripheral zone cancer			
	ERG‐/SPINK1−	ERG‐/SPINK1+	*P*‐value	ERG‐/SPINK1−	ERG‐/SPINK1+	ERG+/SPINK1−	*P*‐value
Case number (%)	75 (80.6)	15 (16.1)	N/A	103 (64.8)	24 (15.1)	32 (20.1)	N/A
Age	64 (60.5‐69)	63 (61‐65.5)	.4478	67 (62.5‐71)	64.5 (61.8‐70)	62.5 (55.8‐68)	.0073
PSA (ng/mL)	7.50 (5.76‐10.57)	8.93 (5.55‐13.95)	.5515	8.48 (6.20‐11.44)	7.05 (5.52‐10.62)	8.14 (6.13‐11.33)	.2686
Tumor volume (cm^3^)	2.30 (0.81‐5.10)	2.90 (2.03‐4.33)	.1499	2.86 (0.82‐5.90)	2.28 (0.56‐3.73)	3.50 (1.33‐5.89)	.3175
pT							
pT2 (%)	54 (72)	9 (60)	.3683	49 (47.6)	12 (50.0)	15 (46.9)	.7971
pT3a (%)	21 (28)	6 (40)		32 (31.1)	5 (20.8)	11 (34.4)	
pT3b (%)	0	0		22 (21.4)	7 (29.2)	6 (18.8)	
Grade Group in RP							
GG 1/2 (%)	57 (76)	11 (73.3)	1	47 (45.6)	14 (58.3)	24 (75)	.0119
GG 3‐5 (%)	18 (24)	4 (23.7)		56 (54.4)	10 (41.7)	8 (25)	
Intraductal carcinoma (%)	7 (9.3)	3 (20)	.361	49 (47.6)	12 (50)	10 (31.2)	.2336
Positive surgical margin (%)	22 (29.3)	4 (26.7)	1	37 (35.9)	8 (33.3)	9 (28.1)	.7544
Lymph node metastasis							
pN0 (%)	67 (89.3)	15 (100)	.7599	88 (85.4)	16 (66.7)	30 (93.8)	.055
pN1 (%)	3 (4.0)	0 (0)		8 (7.8)	4 (16.7)	2 (6.2)	
pNx (%)	5 (6.7)	0 (0)		7 (6.8)	4 (16.7)	0 (0)	

Note

Continuous variables are shown in median value and interquartile range.

### 3.5 Clinicopathological significance of ERG/SPINK1 status in peripheral zone cancers

In the PZ cancer group, a significant difference was not shown for the BCR‐free survival rate among the three subgroups, ERG‐/SPINK1‐, ERG‐/SPINK1+, and ERG+/SPINK1‐ (*P* = .39, Figure 2b). Meanwhile, in comparing clinicopathological factors, the distribution of GG showed a significant difference (*P* = .0119, Table 5). The proportion of high GG (GG3‐5) cases was 54.4% in the highest ERG‐/SPINK1‐ subgroup, 41.7% in the second highest ERG‐/SPINK1 + subgroup, and 25% in the lowest ERG+/SPINK1‐subgroup. Other clinicopathological parameters excluding patient age did not show any significant differences.

## 4 DISCUSSION

We have previously shown the pathological differences in RP specimens between TZ and PZ cancers.^9^ In this study, we conducted a comprehensive analysis to examine the association between clinical prognosis and tumor location in a larger cohort adopting stricter definitions of TZ and PZ cancers. We observed that the prevalence of TZ cancer was lower than that noted in our previous report (42%) because of the stricter definition of tumor location and cohort expansion. Further, patients with TZ cancer showed significantly better prognoses in terms of both BCR and CP. Several studies have shown similar results regarding BCR.^8^, ^11^, ^26^, ^27^ However, to the best of our knowledge, the current study is the first to analyze the association between tumor location and CP, particularly considering that CP is a more significant parameter than BCR because it has a more direct association with life expectancy. None of the 93 cases of TZ cancer markedly developed CP for 8 years of the median follow‐up period, which may provide a great impact on clinical practice such that TZ cancer patients may be able to opt for less invasive treatment strategies in the future.

Regarding pathological features, TZ cancers showed a lower grade and cT/pT stage. In our previous study, tumor grade in RP specimens did not show a significant difference according to tumor locations. This disparity may be explained by the larger cohort in the current study and adoption of a stricter definition of tumor location to exclude ND cases from this study's analyses. Similar results have been reported irrespective of nations and regions, in which TZ cases showed a lower grade and T stage.^7^, ^11^, ^21^, ^26^, ^28^ In particular, the tumor volume in the RP specimen in this study did not show any significant differences according to tumor locations, although PZ cancers showed a significantly higher percentage of the positive core. At the time of this cohort, conventional transrectal biopsy with 10‐12 cores was mainly adopted and targeted and/or transperineal saturation biopsy was rarely performed. Therefore, this disparity can be anatomically explained in that the TZ is more distant from the rectal wall than the PZ. Overall, our results highlight significant differences in prognoses and clinicopathological features between TZ and PZ cancers, and these should be applied in the prostate cancer management.

Here, we examined the immunohistochemical features of TZ and PZ cancers using three antibodies, ERG, PTEN, and SPINK1. The TZ cancer cases showed a significantly lower prevalence of ERG‐overexpression and PTEN‐loss. However, the prevalence of SPINK1 expression did not significantly differ according to the two tumor locations. Regarding the relationship between ERG/SPINK1 expression and clinicopathological parameters, among TZ cancers, ERG‐/SPINK + cases showed a significantly lower BCR‐free survival rate than ERG‐/SPINK1‐ cases. In contrast, among the PZ cancer cases, ERG‐/SPINK1‐ cases showed the highest GG (*P* = .0119). The prevalence of PTEN‐loss, which was associated with a lower BCR‐free survival rate, was significantly higher in the PZ cancer cases. These results of PTEN status suggest that the difference in the prevalence of PTEN‐loss caused prognostic differences between two tumor locations. Several studies have shown the lower prevalence of TMPRSS2/ERG gene fusion/ERG‐overexpression in TZ cancer cases.^17^, ^29^, ^30^ PTEN‐loss was reportedly associated with a poorer prognosis, although the prevalence according to different tumor locations has not been examined.^31^ Regarding the clinicopathological aspect of ERG/SPINK1 expression, SPINK1 expression has been reportedly associated with a worse outcome among ERG‐negative PCa.^19^, ^20^ However, our results suggest that the clinicopathological significance of ERG/SPINK1 status is different between TZ and PZ cancer cases, thereby suggesting the presence of different genetic alterations in the carcinogenesis and progression in both types of cancers. However, further examination is necessary to confirm this hypothesis.

We showed a variety of clinicopathological differences between TZ and PZ cancers, including novel findings in the prognosis of CP and immunohistochemical features. These findings will provide important implications for diagnosing and treating PCa. The TZ cancers were shown to be associated with a lower detection sensitivity by Prostate Imaging‐Reporting and Data System, Version 2,^32^ the golden standard of PCa diagnosis by MRI worldwide.^33^ The technical advances in the imaging diagnosis and biopsy method, including MRI‐ultrasound fusion targeted biopsy, will allow for more accurate preoperative diagnoses of tumor location. This will lead to a more affirmative adoption of less invasive treatments such as active surveillance and focal therapy for TZ cancers, which have more indolent clinicopathological features and a better prognosis. Moreover, observations could be adopted more commonly to elderly TZ cancer patients as a treatment option. In contrast, this approach should be adopted more carefully for PZ patients. However, this study has several limitations including its relatively small number of cases due to its single institute design, selection bias for RP as a treatment option, and an old cohort from approximately 10 years ago. Because of the small cohort size, available statistical analysis methods were limited. Considering the recent advances in techniques for PCa diagnosis, cancer biology of this cohort could be different from recent cases at the diagnostic stage. Further investigations need to be performed with a multi‐institutional cohort incorporating novel diagnostic technologies including MRI and targeted biopsy.

In conclusion, TZ cancers showed better outcomes and different clinicopathological features than PZ cancers. Thus, TZ cancers may be treated more conservatively.

## CONFLICT OF INTEREST

None declared.
